# Engineering climate-resilient horticultural crops: advances in transcriptional regulation, genome editing, and synthetic networks

**DOI:** 10.1093/hr/uhag119

**Published:** 2026-04-02

**Authors:** Wenkang Wu, Rong Wang, Jiaxin Li, Zhen Zhang, Wenkong Yao, Ningbo Zhang, Weirong Xu

**Affiliations:** School of Enology and Horticulture, Ningxia University, Yinchuan, Ningxia 750021, China; School of Enology and Horticulture, Ningxia University, Yinchuan, Ningxia 750021, China; School of Enology and Horticulture, Ningxia University, Yinchuan, Ningxia 750021, China; School of Enology and Horticulture, Ningxia University, Yinchuan, Ningxia 750021, China; School of Enology and Horticulture, Ningxia University, Yinchuan, Ningxia 750021, China; School of Enology and Horticulture, Ningxia University, Yinchuan, Ningxia 750021, China; Engineering Research Center of Grape and Wine, Ministry of Education, Ningxia University, Yinchuan, Ningxia 750021, China; Key Laboratory of Modern Molecular Breeding for Dominant and Special Crops in Ningxia, Yinchuan 750021, China; School of Enology and Horticulture, Ningxia University, Yinchuan, Ningxia 750021, China; Engineering Research Center of Grape and Wine, Ministry of Education, Ningxia University, Yinchuan, Ningxia 750021, China; Key Laboratory of Modern Molecular Breeding for Dominant and Special Crops in Ningxia, Yinchuan 750021, China; State Key Laboratory of Efficient Production of Forest Resources, Yinchuan 750021, China

## Abstract

Abiotic stresses—particularly cold, drought, and salinity—pose significant threats to the productivity and sustainability of horticultural crops. Recent studies have revealed conserved and species-specific regulatory mechanisms that allow plants to adapt dynamically to these environmental constraints. This review synthesizes advances in understanding key transcription factor families—such as CBF/DREB, NAC, MYB, WRKY, and bHLH—that orchestrate stress-responsive gene networks and modulate physiological processes, including osmotic regulation, antioxidant defense, and ionic homeostasis. We also discuss the emerging roles of chromatin remodeling, DNA methylation, histone modifications, and noncoding RNAs in conferring transcriptional plasticity and stress memory. Beyond endogenous pathways, we evaluate transgenic strategies, CRISPR/Cas-based genome editing, and synthetic gene circuits for engineering abiotic stress tolerance. Particular attention is given to trade-offs between growth and defense, challenges in horticultural crop transformation, and gaps in field translation. We further examine the regulatory role of secondary metabolites—such as flavonoids and salicylic acid—as biochemical interfaces between signal transduction and adaptive responses. Finally, we propose a forward-looking roadmap integrating multi-omics, ideotype design, and precision breeding toward climate-resilient horticultural systems.

## Introduction

Climate change is profoundly reshaping global agriculture, threatening the stability of food systems worldwide. While staple crops provide caloric security, horticultural crops are the pillars of nutritional security and economic vitality. However, their productivity and marketable quality are disproportionately vulnerable to the increasing frequency of abiotic stresses, particularly cold, drought, and salinity. Unlike grains, where biomass is the primary output, horticultural commodities rely on intricate physiological balance to maintain texture, flavor, and visual appeal, all of which are easily compromised by cellular homeostasis disruption and oxidative stress [1]. Consequently, developing resilient cultivars that maintain both yield and quality under erratic climate conditions is not merely an agronomic goal but a fundamental necessity for sustainable horticulture.

Horticultural species exhibit remarkable evolutionary divergence in their survival strategies. Annual herbaceous crops, such as pepper (*Capsicum annuum*) and tomato (*Solanum lycopersicum*), predominantly rely on stress avoidance or rapid life-cycle completion [2]. In stark contrast, woody perennials like grape (*Vitis vinifera*) deploy complex, seasonally orchestrated programs—such as endodormancy and cambial cessation—to survive winter freezing [3]. This dichotomy between the transient physiological adjustments of annuals and the deep, cyclical reprogramming of perennials presents unique challenges and opportunities for characterizing molecular resilience. Furthermore, domestication has often inadvertently narrowed the genetic diversity of these crops, selecting for yield at the expense of ancestral stress-adaptive mechanisms [4, 5].

Recent strides in multiomics have begun to map the regulatory landscapes underpinning these adaptations. Central to these networks are hierarchical TF modules—including the CBF/DREB, MYB, bHLH, and NAC families—which coordinate the trade-off between growth and defense [6, 7]. Superimposed on this transcriptional layer are epigenetic mechanisms, such as histone modification and DNA methylation, which confer transcriptional plasticity and ‘stress memory’—a feature particularly critical for long-lived perennials exposed to recurrent seasonal stresses [8, 9].

Despite this mechanistic progress, a translational chasm remains. Most insights are derived from model plants, and their application in horticultural crops is hindered by complex polyploid genomes, recalcitrance to transformation, and the frequent occurrence of ‘yield penalty’ when stress genes are constitutively overexpressed. Here lies the transformative potential of emerging biotechnologies. The transition from descriptive biology to rational engineering—enabled by CRISPR/Cas-mediated precision editing and synthetic gene circuits—offers unprecedented precision to rewrite stress response [10]. These tools allow us to move beyond simple gene overexpression toward the design of ‘smart’ crops with dynamic, environment-responsive regulatory networks.

This review synthesizes recent advances in engineering climate resilience in horticultural crops, focusing on three interrelated domains: (i) the specific transcriptional and genetic logic governing responses to cold, drought, and salinity; (ii) the distinct regulatory architectures of key transcription factor (TF) families in annual versus perennial systems; and (iii) the frontier of biotechnological interventions, from base editing to synthetic logic gates. By integrating mechanistic insights with cutting-edge engineering strategies, we propose a forward-looking roadmap for evolving horticultural breeding from chance selection to precision design.

## Abiotic stress signaling and molecular pathways

Plants are constantly exposed to environmental fluctuations, and their ability to perceive, process, and adapt to abiotic stress is orchestrated by a highly integrated signaling network [11]. Stress responses to cold, drought, and salinity are characterized by both stimulus-specific and shared signaling components that converge to coordinate gene expression, cellular defense, and physiological adaptation [12, 13]. This section provides a mechanistic overview of the core signaling modules, key transcriptional regulators, and cross-talk mechanisms that form the molecular foundation of stress perception and response.

### Core signal transduction modules: from perception to integration

**Figure 1:**
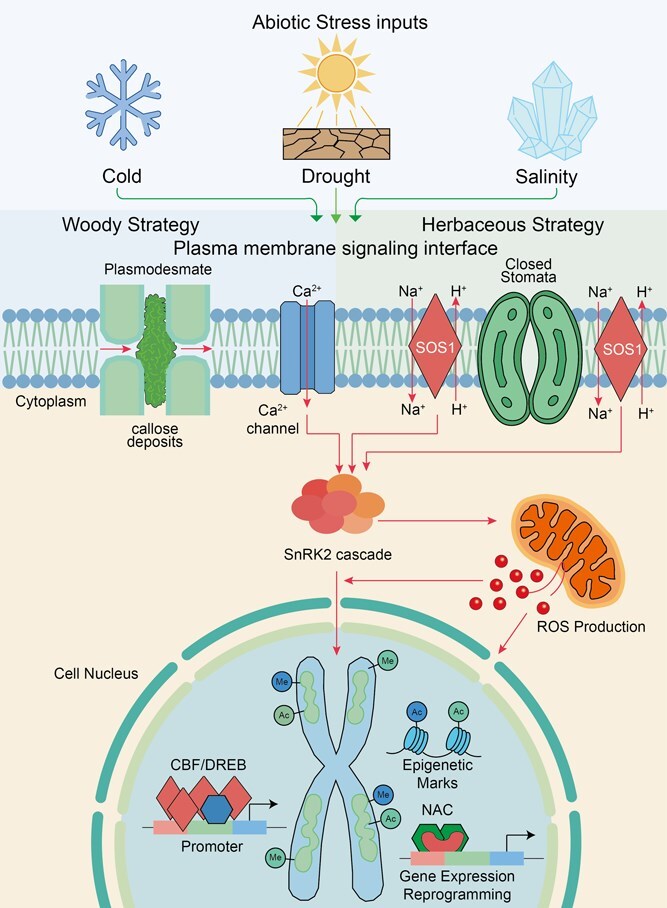
Schematic representation of abiotic stress signaling networks and adaptive strategies in horticultural crops. This diagram illustrates the general signaling pathways activated in plants by abiotic stresses such as cold, drought, and salinity. Abiotic stress inputs are perceived at the plasma membrane interface by specific sensors. For example, the OSCA1/Ca^2+^ channel is activated to mediate the influx of Ca^2+^ into the cytoplasm. Concurrently, RBOHs, located at the plasma membrane interface, generate apoplastic ROS (ROS waves) that function as rapid signal transducers, while distinct mitochondrial ROS (mROS) are generated via the electron transport chain during severe metabolic disruption [15]. These initial calcium and ROS signals converge on the SNF1-related protein kinase 2 (SnRK2) cascade. The activated SnRK2 cascade then relays the signal into the Cell Nucleus, leading to changes in gene expression through TFs like CBF/DREB and NAC, as well as through modifications of epigenetic marks on the chromatin, ultimately resulting in gene expression reprogramming for stress tolerance. The diagram also shows other membrane components like SOS1 transporters (involved in Na^+^/H^+^ exchange) and plasmodesmata channels.

As illustrated in the macroscopic signaling overview in Fig. 1, abiotic stress triggers a rapid and layered signaling cascade, initiating at the plasma membrane and propagating to the nucleus. The primary perception involves specific sensors and ion channels—such as the hyperosmolality-gated calcium-permeable channel OSCA1—which convert physical stimuli into intracellular chemical signals. Following perception, transient cytosolic calcium spikes are decoded by calcium-binding proteins (e.g. calmodulin, CDPKs), which activate downstream kinases and transcriptional responses. Closely coupled with calcium signaling is the rapid production of reactive oxygen species (ROS). While historically viewed as damaging byproducts, ROS generated largely by plasma membrane-localized NADPH oxidases, also known as respiratory burst oxidase homologs (RBOHs), function as critical signal transducers [14]. Under cold and drought stress, these ROS waves interact with calcium signals to activate antioxidant pathways and redox-sensitive TFs.

As stress persists, hormonal signaling integrates these rapid molecular events to sustain adaptation. Abscisic acid (ABA) emerges as a central integrator, particularly under drought and salinity. The core ABA signaling module—comprising Pyrabactin Resistance/PYR1-Like (PYR/PYL) receptors, protein phosphatase (PP2C), and SnRK2 kinases—orchestrates stomatal closure, osmotic adjustment, and stress-responsive gene expression via the phosphorylation of ABA-responsive element-binding factor/ABA-responsive element-binding protein (ABF/AREB) TFs [16, 17]. This pathway is highly interconnected with other hormonal axes. SA has emerged as a pivotal biochemical interface coordinating responses to both biotic and abiotic constraints. Within the integrated signaling network, SA acts as a master regulator of the antioxidant defense system, enhancing the activities of catalase (CAT), superoxide dismutase (SOD), and peroxidase (POD) to scavenge ROS waves triggered by salinity and heat stress (HS) [18]. Crucially, SA engages in sophisticated crosstalk with the ABA-hydraulic nexus; while ABA mediates rapid stomatal closure, SA can antagonistically promote root system architecture (RSA) expansion—via the modulation of gravitropism and lateral root density—to facilitate moisture capture from deeper soil layers under moderate water deficits [18].

Furthermore, exogenous SA application has been shown to stabilize cell walls and reinforce the periderm in perennials, mitigating the cellular lipid peroxidation that leads to frost injury or postharvest chilling symptoms [18]. This SA–ROS–ABA axis forms a flexible decision-making interface, allowing plants to maintain metabolic performance and protect fruit set even under the multifactorial stress combinations prevalent in contemporary climates [19]. For instance, SA—traditionally associated with biotic stress—has been implicated in modulating ROS homeostasis and ABA sensitivity, effectively fine-tuning the trade-off between growth and stress responsiveness [20, 21]. Through this integrated network, diverse inputs at the plasma membrane converge to regulate central transcriptional programs in the nucleus.

### Transcription factors as central regulators

TFs serve as key molecular hubs that integrate upstream signaling cues and orchestrate downstream gene expression programs essential for abiotic stress adaptation. Among them, the CBF/DREB family remains the most extensively studied in the context of cold stress [22]. *C-repeat binding factor 1/2/3* (*CBF1*, *CBF2*, and *CBF3*) regulate a broad suite of cold-responsive (*COR*) genes involved in membrane stabilization, osmotic adjustment, and cryoprotection [23]. However, the efficacy of this canonical regulon varies profoundly among horticultural lineages. In chilling-sensitive species such as tomato, the CBF signaling pathway exhibits functional attenuation; despite the presence of CBF orthologs, the downstream regulon is significantly contracted compared to freezing-tolerant species, activating a limited subset of *COR* genes that fails to confer freezing tolerance [24, 25]. This contrast highlights the evolutionary divergence in regulatory wiring between annual vegetables and hardy perennials [26].

**Figure 2:**
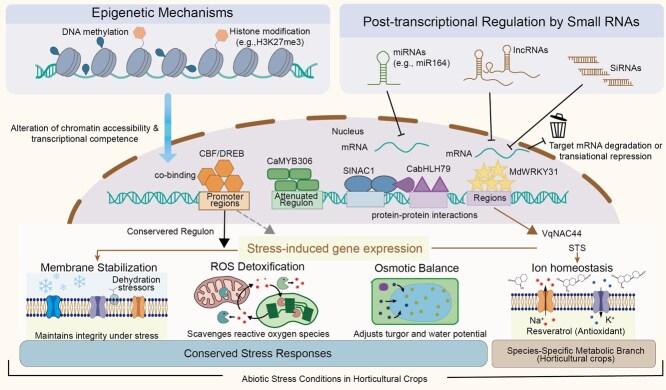
Conceptual model of multilayered regulatory control of abiotic stress responses in horticultural crops. This model depicts the hierarchical regulatory framework underlying abiotic stress-responsive gene expression in horticultural species. Upstream regulation involves epigenetic control through DNA methylation and histone modifications, together with small RNA-mediated post-transcriptional silencing by microRNAs (miRNAs), long noncoding RNAs (lncRNAs), and small interfering RNAs (siRNAs). These signals converge on nuclear TF modules, including CBF/DREB and species-associated regulators such as SlNAC1, CaMYB306, VqNAC44, CabHLH79, and MdWRKY31, which fine-tune gene expression through promoter targeting, co-binding, and protein–protein interactions. The resulting transcriptional outputs support both conserved stress responses and crop-specific metabolic adaptations, including membrane protection, ROS scavenging, osmotic regulation, ion balance, and secondary metabolism.

Beyond the CBF core, other TF families drive species-specific strategies, often linking stress survival with quality traits. The MYB family exhibits context-dependent roles: in pepper, *CaMYB306* acts as a negative regulator of cold tolerance by suppressing antioxidant gene expression [27], whereas other MYB members enhance flavonoid biosynthesis and redox homeostasis [28, 29]. Similarly, NAC family shows unique adaptation in woody fruit crops. In grape, *VqNAC44* diverges from the osmotic-centric function seen in monocots (e.g. rice) by directly regulating *stilbene synthase* (*STS*) genes [30]. This recruits the synthesis of resveratrol as a biochemical shield against oxidative stress. Additionally, *CabHLH79* in pepper activates cold-inducible genes involved in morphological adaptation [31]. Table 1 summarizes the major TF families involved in abiotic stress responses in horticultural crops and their representative functional roles. Notably, these TFs often operate within combinatorial modules, engaging in cobinding at promoter regions or forming protein–protein complexes that enable fine-tuned and context-specific transcriptional responses [32]. Importantly, TF activity is highly dynamic and subject to modulation by stress intensity, developmental stage, and tissue specificity [32]. This layered regulatory architecture—illustrated in Fig. 2**—**integrates TF networks, chromatin-level modulation, and small RNA-mediated post-transcriptional regulation. While extensively characterized in model plants, these interconnected layers remain largely unexplored in perennial horticultural species.

**Table 1:** Divergent roles of key TF families in major horticultural crops

TF family	Representative gene	Species	Stress response	Functional characterization	References
CBF/DREB1	SlCBF1	Tomato	Cold, drought	Induces *COR* genes; regulates postharvest chilling sensitivity	[24]
	VvCBF4	Grape	Cold	Enhances membrane stability and modulates dormancy induction	[33, 34]
	StDREB1	Potato	Salt	Activates proline biosynthesis and mitigates osmotic stress	[35]
NAC	SlNAC1	Tomato	Drought, salinity	Root-specific expression enhances drought tolerance through ABA signaling	[36]
	VqNAC44	Grape	Cold/biotic	Directly activates *STS* for resveratrol production	[30]
	StNAC2	Potato	Heat/salt	Enhances tolerance to heat and salt stress via upregulating antioxidant enzyme expression	[37]
WRKY	MdWRKY31	Apple	Cold/drought	Integrates ABA signaling with sugar metabolic reprogramming	[38]
	CaWRKY27	Pepper	Heat/disease	Positive regulator of thermotolerance and immune defense	[39]
	SlWRKY51	Tomato	Cold	Regulates ROS scavenging through the proline-ascorbate cycle	[40]
MYB	MdMYB1	Apple	UV/cold	Drives anthocyanin accumulation and photoprotective pigments	[41]
	CaMYB306	Pepper	Cold	Acts as a negative regulator and suppresses antioxidant defenses	[27]
	SlMYB306-like	Tomato	Salt	Modulates photosynthesis efficiency and stomatal dynamics	[42]

### Integration of stress signaling pathways

In natural environments, plants rarely encounter abiotic stresses in isolation. Instead, their signaling systems are inherently modular and highly interconnected, allowing for dynamic reconfiguration in response to simultaneous or sequential stress cues. This capacity for network-level integration enables plants to fine-tune physiological responses and allocate resources effectively under complex environmental conditions.

Multiple studies highlight the shared utilization of ABA and reactive ROS signaling pathways across cold, drought, and salinity stress [43, 44]. These common pathways form the molecular basis for cross-tolerance and anticipatory defense strategies. For instance, in trifoliate orange (*Poncirus trifoliata*), transcriptional interaction between CBF1 and ZAT12 exemplifies convergence between cold and drought response networks, suggesting coordinated regulatory outputs [45]. Similarly, in apple (*Malus domestica*), members of the CBF and NAC TF families have been shown to coregulate stress-responsive modules, establishing layered transcriptional hierarchies that respond to diverse and overlapping stimuli [46]. In addition to classical stress hormones, ethylene (ET) and jasmonic acid (JA)—traditionally associated with biotic stress responses—are increasingly recognized as active participants in abiotic stress signaling [47]. In potato and pepper, JA signaling modulates cold and drought tolerance by interacting with ABA-dependent pathways and redox signaling nodes, further expanding the complexity and plasticity of the hormonal network [48]. This intricate hormonal–ROS–TF axis serves as a flexible decision-making interface, allowing plants to prioritize, coordinate, and balance stress responses across gradients of severity, duration, and developmental timing.

## Cold stress tolerance in horticultural crops

Cold stress represents a major constraint on the geographical distribution, phenology, and productivity of horticultural crops. Crucially, the nature of this stress varies by lineage: tropical-origin crops (e.g. tomato, pepper) suffer from chilling injury (0–15°C) characterized by metabolic dysfunction and membrane phase transitions, whereas temperate perennials (e.g. grape, apple) must withstand freezing injury (<0°C) through ice nucleation control and dormancy [49, 50]. To mitigate these effects, plants initiate a cold acclimation response characterized by transcriptional reprogramming, metabolic remodeling, and epigenetic plasticity [51]. Table 2 summarizes the distinct physiological and regulatory features that differentiate chilling injury in tropical annuals from freezing injury in temperate perennials.

**Table 2:** Comparison of cold stress responses between tropical annuals (chilling injury) and temperate perennials (freezing injury)

Features	Chilling injury	Freezing injury
Temperature range	0–15°C	<0°C
Typical crop types	Tropical/subtropical annuals (e.g. tomato, pepper)	Temperate woody perennials (e.g. grape, apple)
Primary cellular damage	Membrane rigidification, metabolic dysfunction, ROS overproduction	Intracellular/extracellular ice crystal formation, severe cellular dehydration
Primary protective strategies	Membrane fluidity maintenance (fatty acid desaturation), compatible solute accumulation (proline, trehalose), antioxidant defense	Control of ice nucleation, physical isolation (callose deposition at plasmodesmata), transition to endodormancy
Transcriptional modules	Transient activation of CBF/DREB regulon (often attenuated or contracted in chilling-sensitive crops)	Integration of cold and photoperiod signals via the Inducer of CBF Expression (ICE)–CBF–DAM module
Epigenetic regulation	Short-term/transient transcriptional priming (e.g. dynamic histone modifications)	Long-term seasonal ‘stress memory’ (e.g. progressive histone H3 lysine 27 trimethylation, H3K27me3 deposition at *DAM* loci serving as a molecular clock)

### Protective modules: from membrane fluidity to structural isolation

Plants deploy a triad of protective strategies that adapt to their specific life histories:

(1) Membrane stability and osmotic buffering

The primary lesion in cold stress is the rigidification of cell membranes. Maintaining membrane fluidity via the desaturation of fatty acids is critical for preventing leakage [52]. Concurrently, the accumulation of compatible solutes—such as proline, trehalose, and raffinose—stabilizes proteins and maintains cellular turgor [53]. In tomato, elevated proline levels are directly linked to enhanced chilling tolerance [40, 54]. *COR* genes, including those encoding LEA proteins and *COR15A*, further contribute to cellular vitrification [55]. While *Arabidopsis* rapidly activates these defenses, species such as potato exhibit delayed induction kinetics, highlighting species-specific thresholds [32].

(2) Structural defense in woody perennials

Distinct from herbaceous annuals, woody perennials employ structural barriers to survive winter. During cold acclimation, species such as grape actively deposit callose at sieve plates and plasmodesmata [32]. This physiological blockade effectively isolates the phloem, preventing the systemic propagation of ice nucleation and inhibiting growth-promoting signals that might prematurely break dormancy [56, 57]. This strategy represents a fundamental evolutionary divergence: physical isolation in perennials versus metabolic adjustment in annuals.

(3) Redox and proteostatic homeostasis

Low temperatures provoke the overproduction of ROS such as H₂O₂ and superoxide, necessitating robust antioxidant defenses [58]. Enzymes such as SOD, POD, and CAT are upregulated in cold-tolerant potato cultivars, mitigating oxidative damage and maintaining redox equilibrium [59].

### Transcriptional regulation: divergence between survival and dormancy

The transcriptional machinery decodes temperature fluctuations into precise gene expression programs. The central hub is the CBF/DREB1 regulon, which orchestrates the activation of *COR* genes [60, 61]. However, the upstream regulation of this conserved module diverges significantly across life histories. In herbaceous crops, *CBF* expression is primarily responsive to acute temperature drops. In contrast, woody perennials integrate CBF signaling with photoperiodic cues to regulate the *Dormancy-Associated MADS-box* (*DAM*) genes. This ICE–CBF–DAM module coordinates the transition from acclimation to endodormancy, a survival strategy unnecessary in annuals [62, 63]. This regulatory layer ensures that meristems remain distinctively quiescent during transient warm spells in winter [64].

Beyond survival, horticultural crops utilize TFs to modulate quality-related traits under stress. In apple and grape, the MYB–bHLH–WD40 (MBW) complex, activated largely through elongated hypocotyl 5 (HY5), drives anthocyanin accumulation-serving dual roles as antioxidants and photoprotective pigments for fruit coloration [65, 66]. Conversely, fine-tuning involves negative regulators; in pepper, *CaMYB306* suppresses antioxidant genes, potentially acting as a brake to prevent resource exhaustion [27, 67].

Other TF families also contribute to these networks. For instance, *SlNAC1* in tomato and its orthologs in other crops confer tolerance through osmotic regulation [50]. Interestingly, potato CBF orthologs exhibit a strong dependency on ABA thresholds, suggesting that in tuber crops, the distinct ‘cold’ and ‘dehydration’ signaling axes have evolutionarily converged [68].

### Epigenetic modulation and stress memory

Epigenetic reprogramming represents a crucial layer of transcriptional control under cold stress. Histone modifications such as *H3K9* acetylation and *H3K27* trimethylation regulate chromatin accessibility at key cold-inducible loci [69–71]. Cold-triggered chromatin decondensation at *CBF* promoters facilitates their rapid activation, while removal of repressive marks at *COR* gene regions supports dynamic transcriptional reprogramming. DNA methylation patterns also shift during cold acclimation [72]. In potato and grape, cold-tolerant genotypes display reduced methylation at stress-responsive promoters, correlating with enhanced gene expression [73]. Noncoding RNAs—including miRNAs and lncRNAs**—**add yet another dimension, modulating mRNA stability, translation, and chromatin architecture [74]. Intriguingly, some epigenetic modifications are heritable or persistent, functioning as a form of stress memory. This ‘primed’ chromatin state enables faster and stronger responses upon recurrent cold exposure [75].

In perennial fruit trees, epigenetic modifications function as a quantitative seasonal timer, enabling plants to distinguish transient temperature fluctuations from stable seasonal shifts. The *Dormancy-Associated MADS-box* (*DAM*) gene cluster, particularly the *DAM5* and *DAM6* loci in peach and apple, serves as the central molecular clock for winter dormancy [76]. This timer operates through an orchestrated shift in chromatin valence: during dormancy induction in late autumn, high levels of permissive H3K4me3 maintain *DAM* expression to suppress growth; as the plant accumulates cumulative chilling, Polycomb Repressive Complex 2 (PRC2) is recruited to the *DAM* gene body, catalyzing the progressive deposition of repressive H3K27me3 marks [77]. This ‘molecular hourglass’ integrates cold recognition over months; once the repressive H3K27me3 density reaches a critical threshold, *DAM* transcription is fully silenced, effectively ‘resetting’ the meristematic competency to respond to growth-promotive signals in the spring [76]. This mechanism prevents ‘false spring’ phenomena—where untimely bud burst during mid-winter warm spells would lead to catastrophic freezing injury—and represents a fundamental evolutionary divergence from the transient transcriptional priming observed in annual crops [78]. Moreover, epigenetic modifications function beyond short-term memory; they serve as a molecular integration of chilling accumulation. For instance, the progressive deposition of repressive H3K27me3 marks at *DAM* loci during winter acts as a seasonal timer, permitting dormancy release only after sufficient cold exposure [64]. This epigenetic ‘molecular clock’ ensures that meristematic activity resumes only under favorable spring conditions, distinguishing the long-term seasonal memory of fruit trees from the transient transcriptional priming observed in annual vegetables.

## Heat stress: thermotolerance and quality maintenance

Global warming poses a direct threat to horticultural productivity, where elevated temperatures often coincide with critical reproductive phases. HS induces widespread protein misfolding and excessive ROS production, and disrupts the metabolic sink–source balance [79]. The core HS response is orchestrated by the heat shock factor (HSF) family. In tomato, *SlHSFA1* acts as a master regulator that rapidly integrates thermal signals to drive the expression of molecular chaperones, including Heat Shock Protein 70 and Heat Shock Protein 90 (*SlHSP70*, *SlHSP90*), which are essential for maintaining the proteostasis of developing anthers and ensuring fruit set under temperatures exceeding 35°C [80].

Beyond survival, HS critically impairs the visual and nutritional quality of horticultural products. In grape and red-fleshed kiwifruit, high temperatures stimulate the accumulation of the endoplasmic reticulum (ER)-stress sensor *VvbZIP17*, which antagonizes *VvFHY3* activity to coordinately repress anthocyanin biosynthesis genes [81]. Simultaneously, HS accelerates the enzymatic degradation of existing pigments via the upregulation of laccase-12-like and class III peroxidases, leading to the rapid fading of floral and fruit coloration [82]. Recent strategies have moved toward engineering ‘thermally buffered’ secondary metabolism, utilizing heat-stable promoters to drive antioxidant enzymes that protect the photosynthetic apparatus from irreversible heat-induced senescence [83].

## Drought and salt stress adaptation mechanisms

Drought and salinity are among the most pervasive abiotic constraints in horticulture, leading to cellular dehydration, oxidative stress, and disrupted ionic homeostasis [84, 85]. Unlike grains, horticultural crops are particularly vulnerable because their economic value relies on turgor-driven fruit expansion and visual quality, which are compromised even by mild stress. Despite their distinct triggers—limited water availability for drought and osmotic/ionic stress for salinity—their molecular responses share substantial overlap, particularly in hormone signaling and redox regulation. This section explores the signaling architectures and adaptive trade-offs governing tolerance.

### Drought stress: the ABA-hydraulic nexus

Drought stress is rapidly perceived through changes in turgor pressure and hydraulic conductance, initiating the synthesis of ABA [64]. Central to this response is the ‘pyrabactin resistance’ Pyrabactin Resistance/PYR1-Like/Regulatory Components of ABA Receptors (PYR/PYL/RCAR) receptor module. Upon ABA binding, these receptors inhibit *PP2C* phosphatases, releasing *SnRK2* kinases. Activated *SnRK2s* not only phosphorylate ABF/AREB TFs to drive gene expression but also directly target membrane channels like *SLAC1* to trigger stomatal closure [86, 87]. Beyond stomatal control, ABA reshapes RSA. In crops like pepper and tomato, ABA signaling promotes hydrotropism—the directional growth of roots toward moisture gradients—and enhances lateral root density to maximize soil exploration [88]. Crucially, this survival response comes at a cost: ABA antagonizes growth-promoting hormones (e.g. cytokinins), deliberately suppressing shoot biomass to conserve water [17]. Balancing this ‘survival-over-yield’ trade-off remains a primary challenge in breeding drought-resilient cultivars.

### Salt stress: ionic homeostasis and tissue partitioning

Salt stress imposes dual pressures: immediate osmotic shock followed by cumulative ionic toxicity (Na^+^ accumulation).

(1) SOS pathway and tissue specificity

The salt overly sensitive (SOS) pathway is the canonical machinery for Na^+^ extrusion [89]. Salt-induced Ca^2+^ spikes activate the SOS3–SOS2 kinase complex, which phosphorylates the plasma membrane Na^+^/H^+^ antiporter *SOS1* to pump Na^+^ out of the cell [90, 91]. Interestingly, horticultural crops exhibit unique spatial deployment of this pathway. While the SOS paradigm is conserved, its spatial deployment differs. In pepper, *CaSOS1* expression is strictly confined to the root epidermis and vascular cylinder [92]. This tissue-specific compartmentalization acts as a ‘gatekeeper’, preventing Na^+^ loading into the xylem and protecting the metabolically active fruit sinks from toxicity—a strategy distinct from the ubiquitous expression seen in *Arabidopsis*.

(2) HKT1 and Na+ unloading complementing SOS1

The high-affinity potassium transporter 1 (HKT1) transporter functions in retrieving Na^+^ from the xylem sap, preventing its transport to shoot tissues [93]. This mechanism is vital for protecting photosynthetic leaves and developing fruits from ionic damage.

(3) ABA–ROS–aquaporin crosstalk

Salt stress stimulates ABA accumulation, which feeds back to upregulate SOS components [94]. Concurrently, ROS (specifically H₂O₂) act as signal transducers. Aquaporins (e.g. plasma membrane intrinsic proteins, PIPs) function as dual conduits for water and H₂O₂, regulated by ABA-mediated phosphorylation [25, 95]. This coregulation maintains a delicate balance between hydraulic conductivity and ROS signaling.

### Transcriptional regulators: orchestrating osmotic and ionic balance

TFs translate signaling cues into adaptive gene expression. (i) Osmotic adjustment (NAC and DREB): The NAC family plays a prominent role in osmolyte biosynthesis. While *OsNAC1* in rice is a classic example, horticultural orthologs perform similar functions. For instance, in tomato, NAC factors activate genes for proline and trehalose synthesis to maintain turgor [96, 97]. (ii) Stomatal and ionic control (MYB and bHLH): The MYB family, such as *CaMYB* in pepper, enhances water-use efficiency by modulating stomatal aperture and oxidative defense. Similarly, *CabHLH79* regulates ion transporters and aquaporins to fine-tune cellular water balance under salt stress [98]. (iii) Signal integration: WRKY TFs, such as *ZmWRKY40*, integrate ABA and ROS signals to modulate cell wall remodeling [99]. These TFs do not act in isolation but form combinatorial modules, allowing plants to mount a tailored response that matches the specific combination of osmotic and ionic pressures.

### Integration: convergence and conflict

Although drought and salinity arise from distinct triggers, they converge on shared signaling hubs (ABA, ROS, Ca^2+^) and overlapping gene networks [100, 101]. This ‘core environmental stress response’ enables cross-tolerance. For example, in *Arabidopsis*, *DREB2A* confers tolerance to both stresses, a mechanism likely conserved in crops [102, 103]. Similarly, in cotton (*Gossypium hirsutum*), the coregulation of aquaporins and antiporters highlights a shared regulon for homeostasis [104, 105]. However, real-world scenarios often involve conflicting signals. For instance, during combined drought and HS, the ABA signal to close stomata conflicts with the cooling requirement to open them. Understanding how plants resolve such antagonistic cues via hierarchical regulatory networks remains a frontier in stress biology [106]. Deciphering these decision-making logics through multiomics and network modeling will be essential for engineering ‘smart’ crops capable of thriving in complex field environments.

## Adaptive mechanisms and multitrait stress resilience

Survival under abiotic stress requires plants to deploy a coordinated suite of morphological, physiological, and metabolic adaptations. Crucially, for horticultural crops, these adaptations must be balanced against the imperative to maintain the visual and nutritional quality of the harvestable organ—be it a fruit, tuber, or leaf [107]. This section outlines the modular structure of stress adaptation, moving from architectural remodeling to the rational design of ‘Ideotypes’ using polyploidy and artificial intelligence (AI).

### Morphological and physiological remodeling

Abiotic stresses induce developmental reprogramming to optimize resource capture [108]. Belowground: RSA is a determinant of tolerance. Drought-adapted pepper cultivars exhibit deeper rooting and enhanced lateral branching to access subsoil moisture [109]. In horticultural systems, this plasticity is frequently engineered via grafting onto resilient rootstocks—a strategy unique to vegetables and fruit trees that allows the scion to inherit the ‘root intelligence’ of wild relatives without genetic introgression [110]. Aboveground: leaf and fruit morphology also adapt. While *Arabidopsis* models show reduced leaf area, horticultural crops prioritize cuticle thickening. For example, in tomato and grape, a robust cuticle not only limits transpiration but also prevents stress-induced fruit cracking, preserving marketability [111]. Woody perennials employ even more permanent defenses, such as the suberization of the periderm and formation of bud scales, creating a physical fortress against winter desiccation distinct from the reversible stomatal closure of annuals [112, 113]. Stomatal control: active regulation via ABA signaling allows for rapid ‘water-saving’ modes [114, 115]. However, this closure restricts CO₂ uptake, creating a fundamental conflict between water conservation and biomass accumulation [116].

### Metabolic adjustments and proteome stabilization

Plants counteract cellular stress through metabolic shifts, but these come with an energetic price tag.

(1) Osmolyte accumulation. The accumulation of proline, trehalose, and glycine betaine buffers osmotic potential and scavenges ROS [117]. However, synthesizing these solutes diverts carbon away from sink organs (fruits/tubers), potentially leading to ‘yield penalty’ or reduced sugar accumulation. Balancing this source–sink trade-off is critical for maintaining fruit quality under stress.
(2) Proteome stabilization. HSPs act as chaperones to prevent protein aggregation [118]. In postharvest contexts, the maintenance of these chaperones is vital to prevent chilling injury symptoms, such as flesh browning in cold-stored fruits [119].
(3) Antioxidant systems. Enzymatic scavengers (SOD, CAT, POD) are upregulated to protect membrane integrity, a trait strongly correlated with freezing survival in potato [32, 120].

### Secondary metabolites in stress mitigation

Secondary metabolites serve as dual-purpose agents, but their high-level induction often triggers a ‘flavor-resilience conflict’ that compromises market value. The core of this conflict lies in metabolic branching points within the phenylpropanoid pathway; for instance, the hyper-accumulation of protective anthocyanins and stilbenes (e.g. resveratrol) during cold or UV stress consumes the majority of available phenylalanine, the common precursor for volatile organic compounds that provide the character-defining ‘floral’ and ‘fruity’ notes in stone fruits and berries [121].

Under escalating HS, the *VvARF3–VvFHY3* module in grapes prioritized survival by suppressing anthocyanin biosynthesis to divert carbon flux toward primary metabolic maintenance, resulting in uneven fruit coloration and reduced antioxidant potential [81]. Similarly, in tomatoes, abiotic stress often enhances organic acid accumulation via upregulated *SlMDH3* activity, which, while beneficial for osmotic buffering, shifts the sugar-to-acid ratio toward a perceived sourness that lacks consumer appeal [122]. Furthermore, stress-induced lignification of the exocarp, intended to prevent dehydration and fruit cracking, can lead to a ‘gritty’ texture that negatively impacts the organoleptic experience of the consumer [123]. Resolving these trade-offs requires the use of tissue-specific synthetic promoters that decouple the defense response in vegetative tissues from the quality pathways in harvestable organs.

### Multitrait integration for stress-resilient ideotypes

Robust resilience is not a single-gene trait but a synergy of structural and metabolic modules [124]. The polyploid advantage: Polyploidization offers an evolutionary fasttrack to resilience. Many horticultural crops (potato, strawberry, banana) are natural polyploids. Polyploid lines demonstrate superior multistress tolerance due to gene dosage effects, increased heterozygosity (buffering), and enhanced epigenetic plasticity [9, 125, 126].

Navigating the complex trade-offs between traits requires new tools. AI and machine learning (ML) are transforming ideotype design. Unlike traditional selection, AI-driven ideotype design leverages advanced convolutional neural networks (CNNs) to decode ‘nonobvious’ morphological traits from high-throughput phenomic datasets. Architectures like DeepLabv3+ utilize atrous spatial pyramid pooling to integrate hierarchical spatial data, allowing for the automated, high-precision segmentation of root systems from complex, nontransparent soil backgrounds where manual error is high [127]. By processing four-channel red-green-blue-depth (RGB-D) images or 3D micro-computed tomography (micro-CT) scans, CNNs can quantify high-dimensional features such as the Gravely-Tropic root angle and precise lateral root insertion patterns—parameters that are functionally linked to subsoil moisture access but are virtually invisible to human breeders [128]. These deep learning models extract multiscale visual features through convolutional layers and use ‘skip connections’ to preserve spatial detail, enabling the prediction of long-term drought recovery rates based solely on seedling-stage root architecture [129].

**Figure 3:**
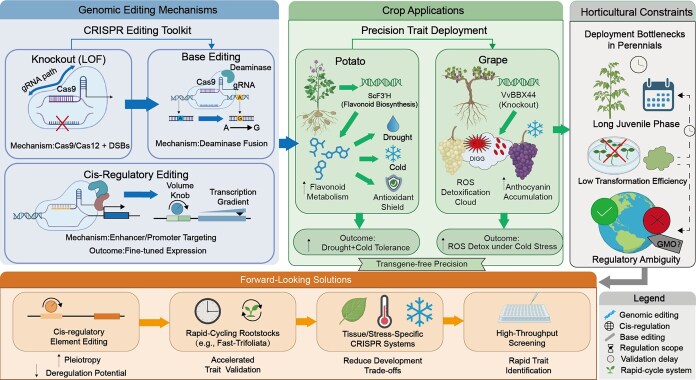
Synergistic integration of natural polyploidy and AI-driven simulation for designing resilient crop ideotypes. A schematic illustrating a convergent framework combining the evolutionary advantages of polyploidy with advanced AI technologies to design multitrait stress resilience in horticultural crops. Left panel: natural polyploidy pathway. Natural polyploid formation confers inherent advantages via gene dosage boost (leading to increased expression), heterozygosity buffering (providing a broad genetic base), and epigenetic plasticity (modulating gene regulation via markers like H3K27me3). These mechanisms contribute to basal tolerance against cold, drought, and salinity in typical polyploid crops (e.g. potato, banana, strawberry). Right panel: AI-driven ideotype simulation. A technological pipeline integrates high-throughput phenotyping and multiomics data (input layer). ML models (e.g. CNNs, random forest) extract nonobvious adaptive trait patterns (ML processing layer). The simulation layer performs in silico trait stacking and predictive modeling to optimize ideotypes and resolve trade-offs between yield and resilience. Center: resilient crop ideotype. Converging inputs facilitate the design of idealized plants (e.g. tomato, grape) characterized by stacked adaptive traits, including root plasticity, osmolyte accumulation, enhanced antioxidant defense, and secondary metabolite production, ultimately achieving stress tolerance while retaining yield and quality. Dashed arrows indicate feedback loops where simulated data refines future AI modeling. A legend for symbols is provided in the bottom right corner. Figure created with Biorender (https://www.biorender.com/).

As illustrated in Fig. 3, robust resilience is not a single-gene trait but a synergy of structural and metabolic modules. Integrating these nonobvious traits with ML-based genomic selection facilitates the ‘predictive stacking’ of physiological ideotypes that break traditional negative correlations between root depth and shoot biomass, accelerating the development of climate-smart horticultural crops [129]. By simulating millions of genetic combinations in silico, AI allows breeders to ‘predictively stack’ physiological traits with metabolic markers, identifying rare ideotypes that break the negative correlation between yield and stress tolerance before a single seed is sown [130, 131].

## Biotechnological approaches for enhancing stress tolerance

Advances in plant biotechnology have transformed the landscape of abiotic stress research—from descriptive studies of signaling networks to the precise, programmable manipulation of gene expression. A new generation of tools, including promoter engineering, CRISPR/Cas genome editing, and synthetic regulatory circuits, enables the targeted reprogramming of stress responses across diverse horticultural species [132, 133]. This section outlines the evolution of these strategies, from constitutive overexpression to rational design.

### First-generation engineering: overexpression and its limitations

Conventional genetic engineering approaches have primarily focused on the constitutive overexpression of stress-responsive TFs or transporters. These strategies aim to broadly reprogram endogenous networks involved in osmotic regulation and detoxification. The CBF/DREB regulon represents the paradigm of this approach. Overexpression of *SlCBF1* in tomato and *LpDREB1A* in diverse hosts significantly enhances cold and drought tolerance through the activation of downstream *COR* genes and osmolyte accumulation [24, 134, 135]. Similarly, the manipulation of ion transporters—notably *SOS1*—has successfully engineered salt tolerance [136]. However, these first-generation strategies often incur a significant ‘yield penalty’ Constitutive activation of stress pathways consumes metabolic energy and disrupts growth hormones, leading to dwarfism, delayed flowering, or yield loss under nonstress conditions [137]. This ‘growth-defense trade-off’ underscores the necessity of moving from ‘always-on’ overexpression to conditional and tunable regulation.

### CRISPR/Cas genome editing: precision without transgenes

**Figure 4:**
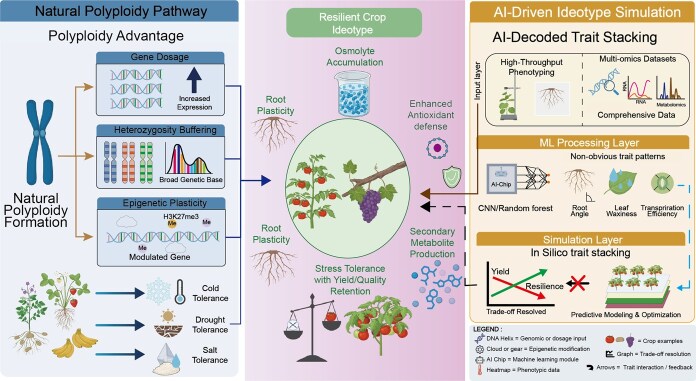
CRISPR/Cas-mediated precision genome editing for abiotic stress resilience in horticultural crops. This schematic illustrates the workflow from editing mechanisms to real-world applications, addressing specific constraints in perennial crops and proposing future strategies for transgene-free trait improvement. Genome editing mechanisms. The CRISPR toolkit utilizes distinct modes: knockout (LOF) via Cas9/Cas12-induced double-strand breaks (DSBs); base editing using deaminase fusions for precise nucleotide conversion (e.g. A to G); and cis-regulatory editing targeting promoters/enhancers for fine-tuned transcriptional regulation without altering coding sequences. Real-world crop applications. Precision trait deployment enables specific metabolic engineering. In potato, targeting *ScF3′H* enhances flavonoid metabolism, conferring drought and cold tolerance. In grape, *VvBBX44* knockout increases anthocyanin accumulation, mitigating ROS accumulation under cold stress. These approaches emphasize transgene-free precision. Horticultural constraints. Deployment in perennial crops is restricted by bottlenecks, including long juvenile phases, low transformation efficiencies, and global regulatory ambiguity regarding genome-edited crops. Forward-looking solutions. Innovative strategies to accelerate trait validation and deployment include *cis*-regulatory editing to reduce pleiotropy, utilizing rapid-cycling rootstocks, developing tissue/stress-specific CRISPR systems, and implementing high-throughput screening platforms. A legend for symbols is provided in the bottom right. Figure created with Biorender (https://www.biorender.com/).

CRISPR/Cas technologies have revolutionized the field by enabling sequence-specific modifications without the permanent integration of foreign DNA. Unlike broad overexpression, CRISPR allows for the precise ‘tuning’ of endogenous loci [138]. Recent applications in horticulture highlight this precision. In potato, targeted editing of *ScF3′H* modulates flavonoid metabolism to improve dual drought–cold tolerance [32]. In grape, knockout of *VvBBX44* releases the suppression of anthocyanin biosynthesis, enhancing oxidative tolerance [139]. A distinct challenge in woody horticulture is the prolonged juvenile phase. Unlike *Arabidopsi*s, where phenotypes are assessed in weeks, validating edits in fruit trees often requires years to reach maturity. This temporal bottleneck necessitates the development of rapid-cycle breeding systems (e.g. using Fast-Trifoliata) to accelerate trait validation. Regulatory landscapes remain a hurdle; while transgene-free edits are deregulated in some nations, others strictly control them [140]. Therefore, editing noncoding regulatory elements (*cis-engineering*) to subtly fine-tune expression represent a promising avenue to bypass both pleiotropic effects and potentially some regulatory concerns (Fig. 4).

### Synthetic biology strategies for stress response engineering

Synthetic biology moves beyond static transgenesis toward the implementation of programmable ‘smart’ gene networks that respond to environmental cues with digital precision. A breakthrough application in horticultural crops is the design of allosteric TF-based biosensors. For instance, the TtgR sensor system can detect internal concentrations of naringenin chalcone, acting as a dynamic metabolic switch that triggers anthocyanin production only when precursors reach a specific threshold, thereby maintaining the precursor pool available for volatile aroma synthesis [141]. Furthermore, the construction of CRISPR interference (CRISPRi)-based Boolean logic gates has enabled the fine-scale programming of RSA. By integrating input signals from localized moisture and nitrogen availability using an ‘AND’ logic gate, researchers have successfully programmed *Arabidopsis* roots to increase branching density only within high-resource soil patches, a strategy now being translated to tomato and citrus to optimize nutrient capture efficiency without constitutive growth penalties [142]. To address the ‘Chromatin Challenge’ in perennial genomes, future synthetic networks will utilize stacked 170-bp insulators to shield these digital circuits from the seasonal waves of H3K27me3, ensuring that the programmed response—such as drought-triggered stomatal closure—remains stable and responsive over the plant’s decades-long lifespan [143].

Synthetic biology offers the ultimate toolkit for decoupling stress tolerance from growth penalties. By designing orthogonal genetic circuits, researchers can program plants to sense and respond to stress with digital precision. Recent breakthroughs include stress-inducible synthetic promoters that drive effector genes (e.g. *CBF*) only under specific conditions, effectively eliminating the yield penalty associated with constitutive expression [144]. More advanced systems employ Boolean logic gates (AND, OR). For instance, a circuit could be designed to trigger stomatal closure only when ‘Drought AND Heat’ signals are detected, mimicking the complex integration seen in nature [145]. To make this concept accessible for traditional horticultural breeding, this ‘AND’ logic gate can be envisioned as a ‘two-key safety vault’. Molecularly, this is often achieved using a split-TF system. For example, a drought-responsive promoter produces one-half of the TF (Key 1), while a heat-responsive promoter produces the other half (Key 2). The target defense gene (the vault) is only activated when both ‘keys’ are present and assemble together under simultaneous drought and heat conditions. This modular precision ensures that energy-consuming defense mechanisms are strictly deployed only when absolutely necessary, effectively eliminating the yield penalties typically seen in conventional overexpression lines [146, 147]. Implementing precision synthetic circuits in perennials faces the ‘Chromatin Challenge’, where the massive seasonal flux of histone modifications (e.g. H3K27me3 enrichment at *DAM* clusters) can lead to unintentional ‘bystander silencing’ or ‘ectopic firing’ of the circuit [148].

Insulators, which are specialized *cis*-regulatory elements, offer a robust theoretical and practical solution to compartmentalize the genome into discrete transcriptional units [143]. These elements function either by physical blocking of enhancer–promoter loops or by establishing metabolic barriers against the spread of repressive heterochromatin [143]. While traditional large insulators like the *Petunia* transformation booster are difficult to integrate into compact synthetic cassettes, recent high-throughput screens using Plant STARR-seq have identified stacks of short 170-bp fragments that retain potent insulator activity across different plant lineages [143]. In mammalian systems, such elements are standard for maintaining consistent transgene expression in differentiating cells; similarly, integrating these ‘genomic shields’ into horticultural gene circuits can provide a stable, buffered environment that protects digital logic (e.g. AND gates) from the dynamic chromatin landscape typical of perennial fruit trees, ensuring functional longevity over multiple growing seasons [149]. However, applying these digital logics to perennial crops faces the ‘Chromatin Challenge’. Seasonal fluctuations in chromatin architecture may lead to the silencing or ‘misfiring’ of synthetic circuits over years—a stability issue unknown in transient assays. Future designs must incorporate ‘insulator’ sequences to protect synthetic modules from the dynamic epigenetic landscape of woody perennials.

### Challenges and future directions

From lab to field: While the toolbox is powerful, the translation of these technologies to horticultural fields faces specific bottlenecks that define the future roadmap. (i) Overcoming transformation recalcitrance: Many elite horticultural cultivars are notoriously difficult to transform. Innovations in developmental regulators (e.g. Wuschel/Baby Boom, WUS/BBM boosters) and nanoparticle-mediated delivery of CRISPR ribonucleoproteins (RNPs) offer routes to bypass tissue culture constraints [150, 151]. (ii) AI-driven design. Predicting the outcome of a genetic intervention in a fluctuating field environment is complex. The integration of multiomics with ML is essential to model genotype × environment (G × E) interactions. ML can guide the selection of optimal editing targets (e.g. which promoter base to change) to maximize resilience while minimizing pleiotropy [152]. (iii) Social and regulatory alignment. Technological success is moot without market acceptance. Transparency regarding the safety of ‘DNA-free’ editing, coupled with clear benefits for the consumer (e.g. reduced pesticide use, better fruit quality), is vital for the commercial adoption of climate-resilient horticultural crops.

## Challenges and future prospects

Despite significant progress in dissecting the molecular frameworks of abiotic stress responses, the translation of mechanistic insights into agronomic resilience remains a bottleneck. Moving forward requires a paradigm shift: from reductionist single-gene studies to systems-level engineering, and from laboratory models to field-proven ideotypes. This section outlines the critical frontiers in bridging this gap.

### Decoupling growth and defense: precision regulation

A persistent challenge in breeding is the fundamental trade-off between stress resilience and agronomic performance [153]. The ‘allocation cost’ theory dictates that constitutive activation of defense pathways—such as the CBF/DREB, MYB, and WRKY regulons—diverts energy from growth, leading to ‘yield penalties’ or delayed phenology under nonstress conditions [154, 155]. For instance, overexpression of *CBF1* improves freezing tolerance but causes severe growth retardation [24]. To resolve these antagonisms, future strategies must focus on ‘dynamic decoupling’. Beyond stress-inducible promoters, precision editing of *cis*-regulatory elements (e.g. upstream open reading frames, uORFs, promoter variants) offers a way to fine-tune translational efficiency rather than acting as a binary switch [156, 157]. Furthermore, engineering protein degradation domains (degrons) into stress TFs could ensure their rapid turnover after stress relief, minimizing metabolic drag.

### From multiomics to pan-genomics

High-throughput omics have revealed complex networks involving osmolyte metabolism and hormone signaling [158, 159]. However, current approaches face limitations in resolution and scope. (i) The pan-genome imperative: Horticultural crops exhibit high heterozygosity and structural variation [160]. Relying on a single reference genome fails to capture the ‘dispensable genome’—the pool of accessory genes often enriched for stress adaptation. Constructing pan-genomes for crops like grape and potato is essential to uncover rare allelic variants lost during domestication [161, 162]. (ii) Spatiotemporal resolution: Traditional bulk RNA-seq masks tissue heterogeneity. Adopting single-cell transcriptomics and spatial profiling will be critical to pinpoint how specific cell types (e.g. stomatal guard cells vs. fruit mesocarp) distinctively respond to stress, enabling more targeted genetic interventions [163]. Developing computational pipelines to integrate these multilayered datasets is essential for converting omics signatures into actionable breeding targets.

### Bridging the lab-to-field gap: the G × E challenge

A major translational chasm exists between controlled environments and the field, particularly for perennials where G × E interactions must be managed over decadal scales. Field failures often manifest as transcriptional dampening, where recurrent seasonal stress triggers the progressive epigenetic silencing of synthetic or ectopic promoters via localized DNA hypermethylation [164]. Another critical failure mode is metabolic exhaustion; long-term studies on transgenic citrus and pear lines demonstrate that constant allocation of resources to ion transporters or osmoprotectants can reduce total nonstructural carbohydrates in storage organs, ultimately compromising floral bud differentiation and fruit set in subsequent years [164].

Furthermore, ‘phenological mismatch’ occurs when engineered stress tolerance disrupts the plant’s intrinsic chilling requirement perception. For example, excessive CBF activity can mask the internal seasonal timer, leading to premature bud break during transient winter warm spells, which exposes vulnerable meristems to late-season frost injury [164]. Bridging this chasm requires shifting from simplistic binary screening to ‘envirotyping’ the high-resolution integration of soil chemistry, microclimate fluctuations, and multiyear metabolic tracking using AI-driven crop simulators to predict long-term performance under unpredictable field trajectories [165].

Genes that confer tolerance in growth chambers often fail under the complex, fluctuating conditions of agricultural ecosystems [166]. This failure stems from genotype × environment interactions, where multiple stressors (e.g. heat + drought + pathogen) occur simultaneously. To overcome this, phenotyping must move from the pot to the plot. High-throughput field phenotyping (using drones and sensors) combined with envirotyping is needed to validate molecular traits in real-world scenarios [167]. Moreover, addressing the consumer acceptance of biotechnological solutions requires a narrative shift: emphasizing that gene-edited crops can reduce resource inputs (water, fertilizer) and ensure food security, thereby aligning technological resilience with ecological sustainability [140, 168].

### Strategic roadmap: integrated design for resilience

Building climate-resilient horticultural systems demands a holistic framework connecting molecular innovation with agronomic reality. (i) Trait pyramiding via AI: The most promising approach involves pyramiding multiple resilience traits into a single ideotype. AI and genomic selection models are transforming this process by predicting the performance of virtual genotypes against future climate scenarios [166]. AI serves as a decision support system to identify optimal combinations of alleles from crop wild relatives—a reservoir of untapped diversity [169]. (ii) Epigenetic priming: In parallel, nontransgenic strategies such as engineering ‘stress memory’ through epigenetic priming offer a durable path to resilience without the regulatory complications of transgene insertion [170, 171]. Ultimately, the future of horticultural breeding lies in precision design—leveraging pan-genomics to find targets, CRISPR/synthetic biology to tune them, and AI to validate them. By aligning these tools, we can develop crops that not only survive the changing climate but thrive to deliver the quality and nutrition the world demands [172].

## Conclusion

Abiotic stress—particularly cold, drought, and salinity—continues to pose a fundamental constraint on horticultural productivity. Unlike staple crops, where biomass is the primary metric, horticultural systems face the unique challenge of maintaining visual and nutritional quality under increasingly erratic climate conditions. Over the past two decades, the field has witnessed a paradigm shift from identifying isolated stress genes to mapping complex, hierarchical regulatory networks. We now understand that adaptive capacity is orchestrated by dynamic hubs involving ABA-ROS-Ca^2+^ signaling, master TFs (e.g. CBF, NAC, MYB), and epigenetic memories that span seasons. This review has synthesized the trajectory of these discoveries, highlighting two critical frontiers. First, the elucidation of species-specific adaptation mechanisms—such as dormancy regulation in woody perennials and fruit-specific osmotic adjustment—has provided precise targets for improvement beyond the generic *Arabidopsis* models. Second, the emergence of biotechnological tools, from CRISPR/Cas genome editing to synthetic logic circuits, offers the unprecedented ability to ‘rewrite’ these stress responses. We are moving from a descriptive era to one of rational design, where stress tolerance can be engineered with minimal penalties to yield or flavor.

However, the path from mechanistic insight to the farm gate remains steep. The persistent trade-offs between growth and defense, the recalcitrance of elite cultivars to transformation, and the regulatory complexities of genome editing require systems-level innovation. Addressing these bottlenecks demands an interdisciplinary convergence: integrating pan-genomics to capture lost alleles, utilizing AI-driven phenomics to validate traits in real-world heterogeneity, and fostering science-policy dialogue to ensure public trust. Looking ahead, the future of climate-resilient horticulture will not rely on single-gene silver bullets, but on the holistic design of ‘smart ideotypes’. By leveraging predictive modeling to stack physiological, metabolic, and architectural traits, we can develop crops that do not merely survive the stresses of the 21st century but thrive to secure the nutritional needs of a changing world.
